# Clearly transparent and air-permeable nanopaper with porous structures consisting of TEMPO-oxidized cellulose nanofibers

**DOI:** 10.1039/d3ra03840h

**Published:** 2023-07-17

**Authors:** Yintong Huang, Takaaki Kasuga, Masaya Nogi, Hirotaka Koga

**Affiliations:** a SANKEN (The Institute of Scientific and Industrial Research), Osaka University 8-1 Mihogaoka Ibaraki Osaka 567-0047 Japan hkoga@eco.sanken.osaka-u.ac.jp +81-6-6879-8444 +81-6-6879-8442

## Abstract

Optically transparent materials that are air permeable have potentially numerous applications, including in wearable devices. From the perspective of sustainable development, 2,2,6,6-tetramethylpiperidine-1-oxyl (TEMPO)-oxidized cellulose nanofibers with widths of 3–4 nm have attracted considerable attention as starting materials for the preparation of clearly transparent nanofiber paper (denoted as conventional nanopaper). However, conventional nanopaper that is prepared from a water dispersion of TEMPO-oxidized cellulose nanofibers by direct drying exhibits poor air permeability owing to its densely packed layered structure. In this study, we prepared a clearly transparent and air-permeable nanopaper by applying filtration-based solvent exchange from high-surface-tension water to low-surface-tension ethanol and hexane, followed by drying under continuous vacuum filtration. The resulting hexane-exchanged nanopaper had a porous structure with individually dispersed and thin nanofiber networks and interlayer pore spaces. Owing to the tailored porous structures, the hexane-exchanged nanopaper provides similar clear transparency (total light transmittance and haze at 600 nm: 92.9% and 7.22%, respectively) and 10^6^ times higher air permeability (7.8 × 10^6^ mL μm m^−2^ day^−1^ kPa^−1^) compared to the conventional nanopaper. This study will facilitate the development of clearly transparent and air-permeable nanopapers to extend their functional applications.

## Abstract 2

Starting with a TEMPO-oxidized cellulose nanofiber/water dispersion, a clearly transparent and air-permeable nanopaper was prepared by filtration-based solvent exchange from water with ethanol and hexane, followed by drying under vacuum filtration.

## Graphical abstract

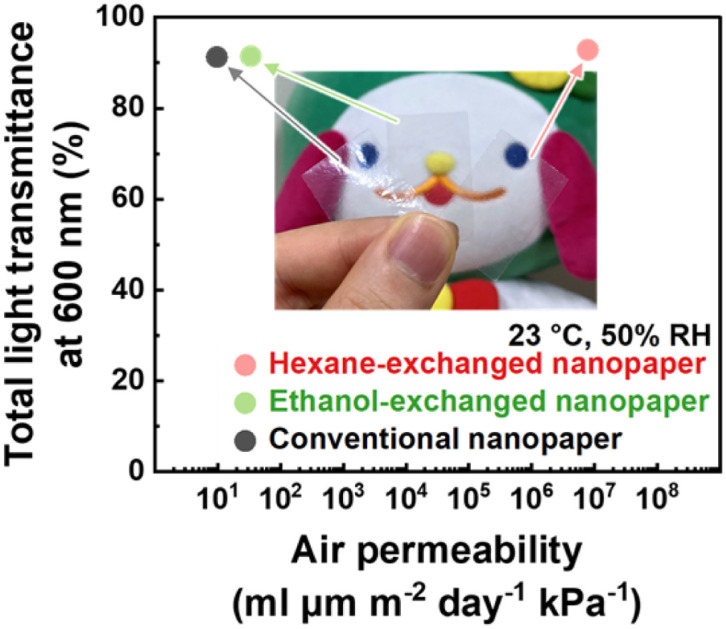

## 1. Introduction

Optically transparent materials, such as glasses and plastics, have been widely utilized for various purposes in everyday life. Recently, transparent materials have been accelerating the development of next-generation wearable electronic^1,2^ and biomedical^3,4^ devices by equipping transparent materials with additional functions such as electrical conductivity,^1^ mechanical stretchability,^2^ and photothermal heating properties.^3,4^ There is a growing demand for incorporating air permeability to transparent materials. This is owing to the limitations of conventional personal-protective equipment, such as face masks and air filters, which lack optical transparency, thus impeding interpersonal communication by hindering the observation of people's expressions during conversations.^5^ Moreover, there is a demand for optically transparent and air-permeable materials to serve as substrates in wearable electronic and biomedical devices to realize visualization of skin surfaces^3,4,6,7^ and wearable comfort for users.^7^

The demand for optically transparent air-permeable materials has prompted extensive research in this field. A promising approach for satisfying this demand involves fabricating porous materials with nanofiber networks. For instance, optically transparent and air-permeable materials have been successfully fabricated by tailoring porous structures with nanofiber networks consisting of polyacrylonitrile nanofibers with widths of ∼200 nm ^8^ or poly(*m*-phenylene isophthalamide)/polyurethane nanofibers with widths of ∼20 nm.^9^ In these porous materials, the use of nanofibers with widths smaller than the visible light wavelength range (360–830 nm) effectively minimizes light scattering, resulting in high optical transparency.^10^ Additionally, the nanofiber network structures with high porosity and small fiber widths facilitate air permeability.^9,11^

Among the various nanofibers, cellulose nanofibers, mainly obtained from wood, tunicates, and bacteria, have attracted increasing attention due to their unique physical properties, abundance, biodegradability, and sustainability.^12–14^ Because cellulose nanofibers exhibit minimal light absorption within the wavelength range of visible light,^15^ possess small fiber widths ranging from 3 to several tens of nanometers,^12,13^ and have high aspect ratios,^12,13^ they hold significant potential for the fabrication of nanofiber-network-structured materials with optical transparency^16–18^ and air permeability.^19,20^ Moreover, cellulose nanofibers possess unique characteristics such as high thermal durability (>180 °C),^21^ low coefficient of thermal expansion (∼6 ppm K^−1^),^13,22^ high strength (∼6 GPa),^23^ and high elastic modulus (∼138 GPa),^13,24^ which further enhance their versatility for use in many emerging applications.^25^ Previous studies have reported the fabrication and air permeability of cellulose nanofiber-based porous materials.^19,20^ For instance, Toivonen *et al.* investigated the optical transparency and air permeability of the cellulose nanofiber aerogel membrane derived from mechanically fibrillated cellulose with widths ranging from 5 to 20 nm and above, with a thickness of ∼25 μm.^26^ However, despite these efforts, achieving enhanced transparency in cellulose nanofiber-based porous materials remains an ongoing area of investigation.

2,2,6,6-Tetramethylpiperidine-1-oxyl (TEMPO)-mediated oxidation of wood pulp, accompanied by mild mechanical disintegration, yields cellulose nanofibers with widths of 3–4 nm.^27,28^ Because of these very small widths, TEMPO-oxidized cellulose nanofibers hold considerable promise as raw materials for the fabrication of clearly transparent porous materials with air permeability. On one hand, reports indicate that TEMPO-oxidized cellulose nanofiber aerogels^29^ and xerogels^30^ exhibit some degree of transparency even with thicknesses of 1 and 6 mm, respectively, although their air-permeability has not yet been assessed. On the other hand, TEMPO-oxidized cellulose nanofibers enable the fabrication of clearly transparent nanofiber paper, also known as nanopaper, with thicknesses in the range of several tens of micrometers.^31–33^ However, conventional TEMPO-oxidized cellulose nanopaper exhibits densely packed structures with low porosities, which contribute to excellent gas-barrier properties (*i.e.*, poor air permeability).^31^ Thus, to the best of our knowledge, the simultaneous achievement of clear transparency and air permeability of TEMPO-oxidized cellulose nanopaper remains challenging.

Herein, we demonstrate the successful fabrication of a TEMPO-oxidized cellulose nanopaper with clear transparency and improved air permeability. The nanopaper prepared from the TEMPO-oxidized cellulose nanofiber/water dispersion by vacuum-filtration drying (conventional nanopaper) had clear transparency but low air permeability because of its dense structure. This study used a filtration-based solvent-exchange process to make the nanopaper porous. The resulting nanopaper with a thickness of ∼22 μm has porous structures with TEMPO-oxidized cellulose nanofiber networks, providing similar optical transparency and considerably higher air permeability than conventional nanopaper.

## 2. Results and discussion

### 2.1 Preparation of TEMPO-oxidized cellulose nanopaper with porous structures

**Fig. 1:**
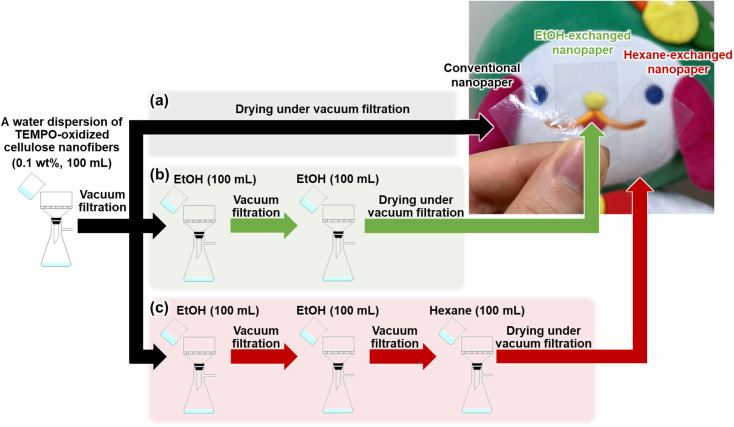
Schematics of preparation procedure and optical images of the (a) conventional nanopaper, (b) EtOH-exchanged nanopaper, and (c) hexane-exchanged nanopaper.

**Fig. 2:**
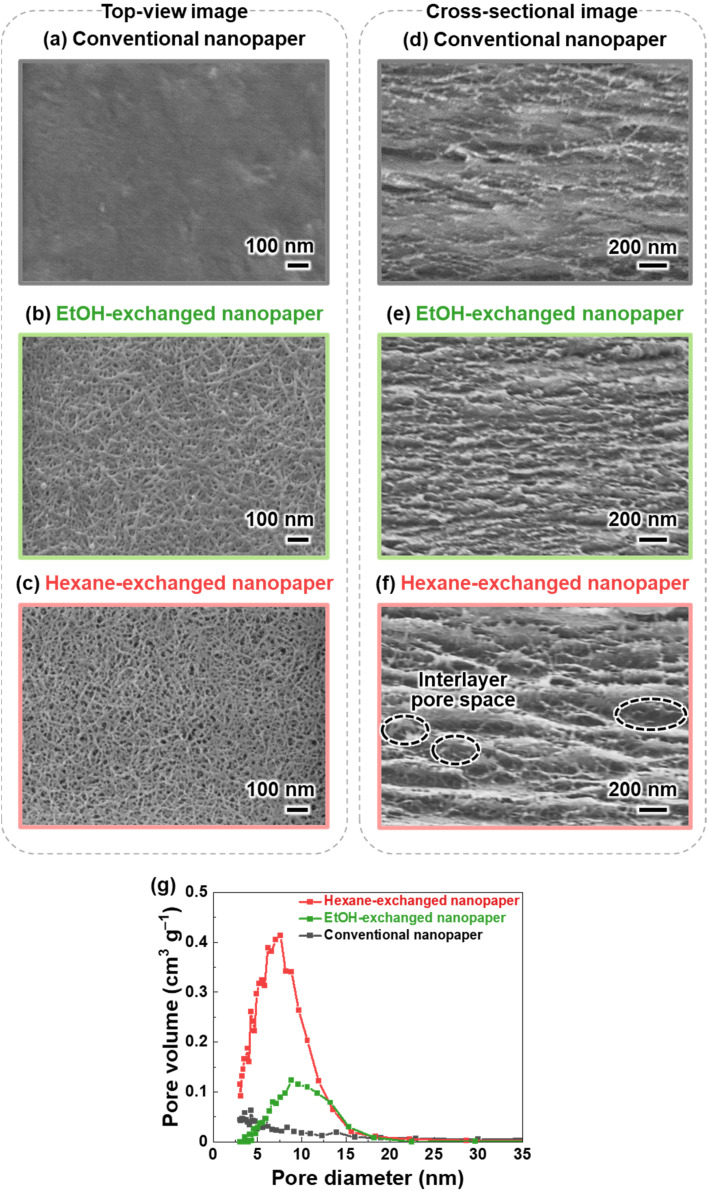
(a–c) Top-view and (d–f) cross-sectional FE-SEM images of the (a and d) conventional, (b and e) EtOH-exchanged, and (c and f) hexane-exchanged nanopapers, (g) pore size distribution curves of the conventional, EtOH-exchanged, and hexane-exchanged nanopapers.

TEMPO-oxidized cellulose nanofibers with a carboxylate content of approximately 1.37 mmol g^−1^ were prepared by TEMPO-mediated oxidization of never-dried softwood bleached kraft pulp,^34^ followed by homogenization by the aqueous counter-collision method.^35^ To prepare conventional nanopaper, a water dispersion of the cellulose nanofibers (0.1 wt%, 100 mL) was first condensed by vacuum filtration. The wet sheet obtained was then dried under further continuous vacuum filtration (Fig. 1a). As seen in the top-view field emission scanning electron microscopy (FE-SEM) image, the as-prepared conventional nanopaper had densely packed surfaces (Fig. 2a). The cross-section FE-SEM image indicated that the conventional nanopaper possessed a paper-like layered structure, with the cellulose nanofibers packed almost parallel to each layer (Fig. 2d), resulting from the forced deposition of the cellulose nanofibers in a water dispersion during vacuum filtration.^36^ Such densely packed layered structure of the conventional nanopaper is attributed to the aggregation of the cellulose nanofibers dispersed in the wet sheet during the drying process due to the capillary force,^37^ which is generated by the high surface tension of water (72.14 mN m^−1^ at 25 °C).^38^

To suppress the aggregation of cellulose nanofibers, filtration-based solvent exchange was conducted before drying. As shown in Fig. 1b, the water dispersion of the cellulose nanofibers was vacuum-filtered, and ethanol (EtOH) with low surface tension (21.93 mN m^−1^ at 25 °C)^38^ was poured onto the resulting wet sheet and then vacuum-filtered. EtOH treatment was repeated twice for sufficient solvent exchange; this was then followed by drying to prepare an EtOH-exchanged nanopaper. From the top-view and cross-sectional FE-SEM images of the EtOH-exchanged nanopaper, the aggregation of cellulose nanofibers in the in-plane direction and the packing of their layers in the through-plane direction were partially suppressed (Fig. 2b and e).

Due to the insufficient suppression of cellulose nanofiber aggregation and interlayer packing during drying with EtOH treatment, further investigation was conducted to exchange the solvent from EtOH to hexane. Hexane with a lower surface tension of 17.90 mN m^−1^ at 25 °C, was chosen^38^ as it exhibited a lower surface tension compared to EtOH. Direct exchange from water to hexane was difficult because of the significant difference in polarity between water (dielectric constant: 80 at 20 °C)^39^ and hexane (with a dielectric constant of 1.9 at 20 °C).^39^ Therefore, a successive exchange from water to EtOH (with a dielectric constant of 24 at 20 °C)^39^ and then to hexane was performed. The hexane-exchanged nanopaper was prepared by drying the resulting wet sheet (Fig. 1c). The top-view and cross-sectional FE-SEM images of the hexane-exchanged nanopaper revealed effective suppression of cellulose nanofiber aggregation in the in-plane direction and layer packing in the through-plane direction. Consequently, the hexane-exchanged nanopaper exhibited porous structures with individually dispersed nanofiber networks and interlayer pore spaces (Fig. 2c and f).

Table 1 lists the thicknesses, bulk densities, and porosities of the conventional, EtOH-, and hexane-exchanged nanopapers. Although all nanopapers were prepared from same starting materials (0.1 wt% and 100 mL of cellulose nanofiber/water dispersion), the EtOH- and hexane-exchanged nanopapers had higher thickness (15.7 ± 1.1 and 21.6 ± 1.6 μm, respectively) and lower bulk density (1.02 ± 0.06 and 0.78 ± 0.06 g cm^−3^, respectively) than the conventional nanopaper (thickness: 12.9 ± 0.5 μm, bulk density: 1.19 ± 0.03 g cm^−3^). These results further suggest that interlayer packing was suppressed by solvent exchange, particularly with hexane. Although the porosity of the nanopaper was increased from 29.4 ± 2.0% to 39.4 ± 3.6 and 53.4 ± 3.8% by the solvent exchange with EtOH and hexane, respectively, all nanopapers had similar pore size distributions (<30 nm) (Fig. 2g). The possible reason can be explained as follows. To prepare all nanopapers, a water dispersion of TEMPO-oxidized cellulose nanofibers was first condensed by vacuum filtration. In this condensed state, cellulose nanofibers are dispersed in the wet sheet; the spaces between the cellulose nanofibers are filled with water retained in the wet sheet. The subsequent solvent exchange from water to EtOH or hexane can suppress closing of the spaces between the cellulose nanofibers by their aggregation during drying. Regardless of this suppression degree, the possible distribution range of pore sizes in the resulting nanopapers (after drying) is determined by the condensed state before the solvent exchange. Thus, the solvent exchange affected the porosity of the nanopaper, rather than its pore size distribution (Fig. 2g and Table 1). Notably, there was no significant difference in the appearance of the nanopapers regardless of their porosity (Fig. 1).

**Table 1:** Structural profiles of the conventional, EtOH-, and hexane-exchanged nanopapers

	Thickness (μm)	Bulk density (g cm^−3^)	Porosity (%)
Conventional nanopaper	12.9 ± 0.5	1.19 ± 0.03	29.4 ± 2.0
EtOH-exchanged nanopaper	15.7 ± 1.1	1.02 ± 0.06	39.4 ± 3.6
Hexane-exchanged nanopaper	21.6 ± 1.6	0.78 ± 0.06	53.4 ± 3.8

### 2.2 Optical transparency

**Fig. 3:**
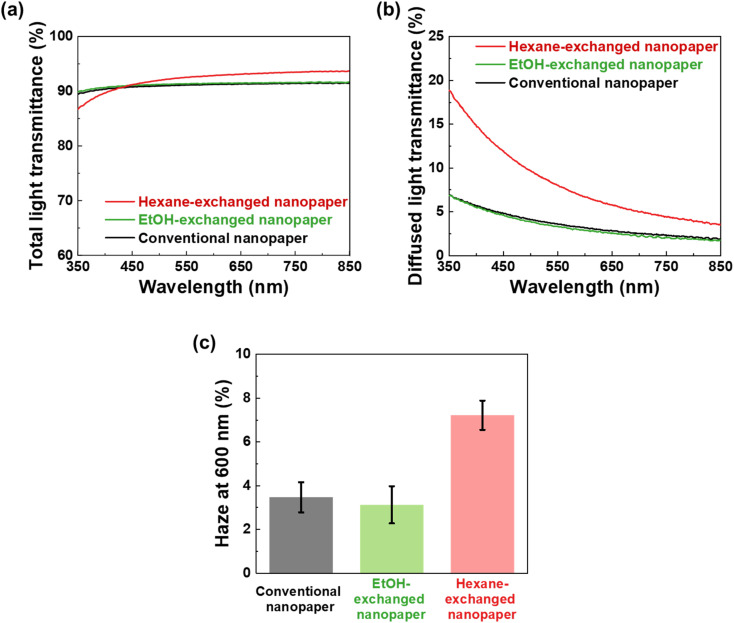
(a) Total light transmittance, (b) diffused light transmittance, and (c) haze value at a wavelength of 600 nm of the conventional, EtOH-, and hexane-exchanged nanopapers at 23 °C and 50% RH.

To further investigate the optical properties of the conventional, EtOH-exchanged, and hexane-exchanged nanopapers, the total light transmittance, diffused light transmittance, and haze values were measured (Fig. 3). Fig. 3a displays the spectra of total light transmittance for the nanopapers. The conventional and EtOH-exchanged nanopapers exhibited a high total light transmittance of approximately 90% within the 350–850 nm wavelength range. Similarly, the hexane-exchanged nanopaper demonstrated a high total light transmittance of approximately 90% for wavelengths longer than 420 nm. However, a slight reduction in total light transmittance was observed for wavelengths shorter than 420 nm in the case of the hexane-exchanged nanopaper. Because the hexane-exchanged nanopaper has a higher porosity than the conventional and EtOH-exchanged nanopapers (Table 1), the incident light on the hexane-exchanged nanopaper is more frequently refracted at the interfaces between the cellulose nanofiber and air. Rayleigh scattering^40^ is considered to be dominant within the hexane-exchanged nanopaper because the width of the TEMPO-oxidized cellulose nanofibers (approximately 3–4 nm)^27,28^ is considerably smaller than the wavelength of visible light (360–830 nm). Rayleigh scattering is facilitated by decreasing the wavelength of incident light (
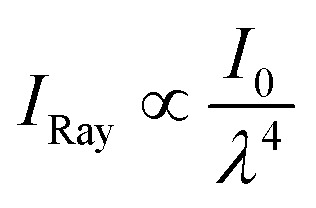
; where *I*_Ray_ is the intensity of Rayleigh scattering, *I*_0_ is the intensity of incident light, and *λ* is the wavelength of incident light).^40^ Therefore, the total light transmittance of the hexane-exchanged nanopaper is slightly reduced at shorter wavelengths (Fig. 3a) due to the Rayleigh scattering phenomenon, which leads to light being reflected to its outer surfaces.

As shown in Fig. 3b, the hexane-exchanged nanopaper exhibited a higher diffused light transmittance than the conventional and EtOH-exchanged nanopapers. The diffused light transmittance of the hexane-exchanged nanopaper increased with decreasing incident light wavelength. This tendency was also consistent with Rayleigh scattering, where the light scattering is facilitated by reducing the wavelength of incident light.^40^ At a typical visible light wavelength of 600 nm, the hexane-exchanged nanopaper demonstrated a haze value (%, 
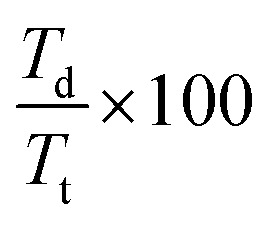
; where *T*_d_ is the diffused light transmittance and *T*_t_ is the total light transmittance) of 7.22%. This value was higher compared to the haze values of the conventional and EtOH-exchanged nanopapers, which ranged from 3.1 to 3.5% (Fig. 3c). However, the hexane-exchanged nanopaper exhibited clear transparency owing to its high total light transmittance (approximately 90%) and low haze value (7.22%). Despite possessing a porosity of over 50%, the hexane-exchanged nanopaper effectively mitigated light scattering through the presence of very-thin-nanofiber network structures shown in Fig. 2c, resulting in clear transparency.

### 2.3 Air permeability

Optically transparent and air-permeable materials have the potential to be used in various applications, including wearable devices. Consequently, the air permeability characteristics of conventional, EtOH-exchanged, and hexane-exchanged nanopapers were evaluated. The air permeability values of the conventional, EtOH-exchanged, and hexane-exchanged nanopapers were determined as 9.7 × 10^0^, 3.4 × 10^1^, and 7.8 × 10^6^ mL μm m^−2^ day^−1^ kPa^−1^, respectively.

The conventional nanopaper demonstrated a low air permeability of 9.7 × 10^0^ mL μm m^−2^ day^−1^ kPa^−1^, as reported for the nanocellulose films.^31,41,42^ This low air permeability can be attributed to the densely packed layered structures derived from cellulose nanofibers (Fig. 2a and d). Such densely packed layered structures increase the tortuosity and diffusion path length for air permeation, consequently hindering air permeation within the nanopaper.^41,42^ Similarly, the EtOH-exchanged nanopaper also exhibited a low air permeability of 3.4 × 10^1^ mL μm m^−2^ day^−1^ kPa^−1^. Although the EtOH treatment partially loosened the densely packed layered structures within the nanopaper (Fig. 2b and e), it was still insufficient to improve the air permeability.

**Fig. 4:**
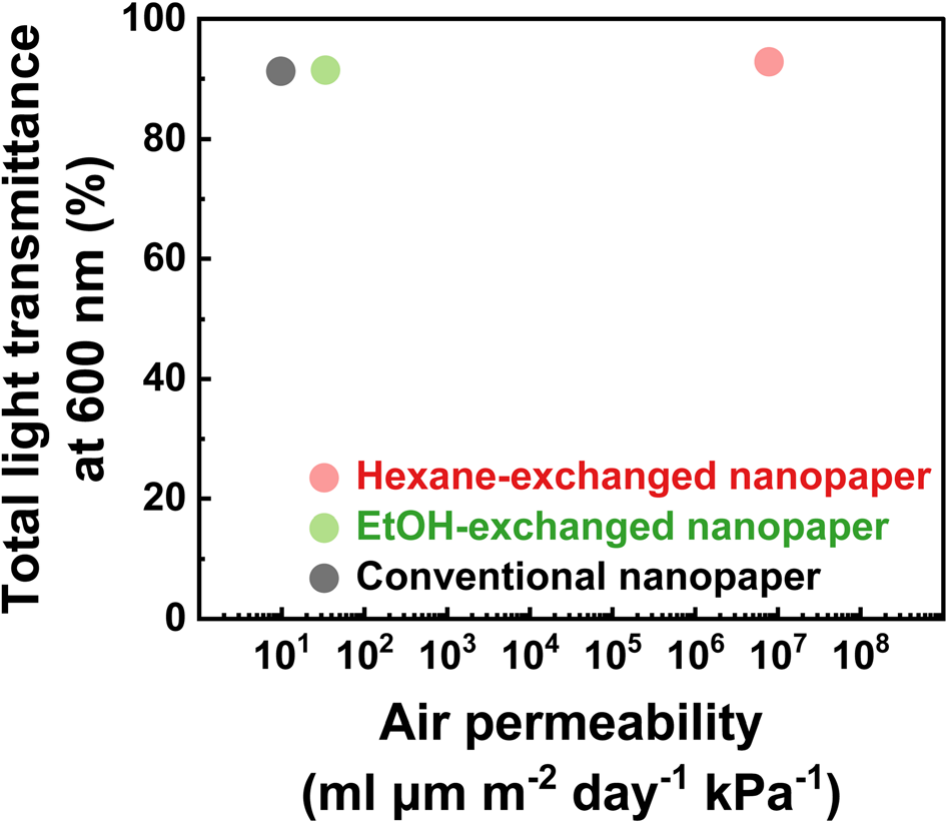
Air permeability *versus* total light transmittance at a wavelength 600 nm of the conventional, EtOH-, and hexane-exchanged nanopapers at 23 °C and 50% RH.

The hexane-exchanged nanopaper exhibited a significant improvement in air permeability (7.8 × 10^6^ mL μm m^−2^ day^−1^ kPa^−1^) compared to the conventional and EtOH-exchanged nanopapers. Because the hexane-exchanged nanopaper had a porous structure with individually dispersed nanofiber networks (Fig. 2c), the Knudsen number could be used to characterize the flow regime of the gas around individual fibers^43,44^ to explain the improved air permeability. The fiber length of fibrous materials has reportedly no significant influence on the through-plane air permeability of the materials as long as the solid volume fraction remains constant.^45^ According to previous reports,^43,44^ the Knudsen number can be calculated for individually dispersed fiber networks in porous media using the following equation:
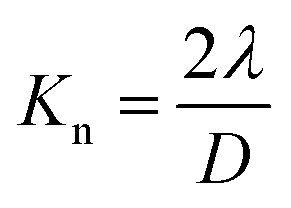
where *K*_n_ is the Knudsen number, *λ* is the mean free path of air molecules (66 nm at normal temperature and pressure), and *D* is the width of fibers. When the Knudsen number exceeds 10, the gas flow regime around the fibers is classified as the free molecular flow regime,^43,44^ where the influence of the fibers on the gas flow field is negligible.^44^ Considering the width of the TEMPO-oxidized cellulose nanofibers, which is approximately 3–4 nm,^27,28^ the Knudsen number for the individual nanofibers within the hexane-exchanged nanopaper can be estimated to be approximately 33–44. Therefore, the hexane-exchanged nanopaper could facilitate air permeation due to its porous structure with individually dispersed and very thin nanofibers. Moreover, the hexane-exchanged nanopaper possesses interlayer pore spaces (Fig. 2f), which reduce the tortuosity and diffusion path length for air permeation,^46^ leading to improved air permeability. Notably, the hexane-exchanged nanopaper achieves 10^6^ times higher air permeability than the conventional nanopaper while maintaining a high level of total light transmittance (92.9% at 600 nm) (Fig. 4).

## 3. Conclusions

In conclusion, a clearly transparent and air-permeable nanopaper was successfully prepared using TEMPO-oxidized cellulose nanofibers. The nanopaper was obtained through a filtration-based solvent exchange process, transitioning from a water dispersion of TEMPO-oxidized cellulose nanofibers to EtOH and hexane, followed by drying under continuous vacuum filtration. The resulting hexane-exchanged nanopaper exhibited a porous structure with a porosity of 53.4% while exhibiting the individually dispersed- and very-thin-nanofiber networks as well as the interlayer pore spaces. These porous structures contributed to the nanopaper's high transparency (92.9% total light transmittance and 7.22% haze at a wavelength of 600 nm) and air permeability (7.8 × 10^6^ mL μm m^−2^ day^−1^ kPa^−1^). While the conventional nanopaper has shown great promise as a green transparent substrate alternative to glasses and plastics, this approach can improve the air permeability of nanopaper while maintaining its transparency to further expand its applicability. Given that cellulose is a widely available and abundant bioresource on Earth, this study also presents a promising pathway for developing clearly transparent, air-permeable, and sustainable materials.

## 4. Experimental

### 4.1 Materials

TEMPO (>98.0% purity) was obtained from Kanto Chemical Industry Co., Ltd, Tokyo, Japan. Sodium bromide (NaBr, >99.5% purity), sodium hypochlorite (NaClO) solution (≧5% available chlorine), 1 M sodium hydroxide (NaOH) solution, EtOH (>99.5% purity), hexane (>95% purity), and other chemicals were purchased from Nacalai Tesque, Inc., Kyoto, Japan. All chemicals were used without further purification.

### 4.2 Preparation of TEMPO-oxidized cellulose nanofibers

TEMPO-mediated oxidation of never-dried softwood bleached kraft pulp was performed, according to a previous report.^34^ Briefly, the pulp fibers (20 g) were suspended in distilled water (2 L); then, a 50 mL of water solution containing 0.32 g of TEMPO and 2 g of NaBr was added to the pulp suspension. TEMPO-mediated oxidation was initiated by adding NaClO (5 mmol per gram of pulp fibers) to the suspension. The pH of the suspension was kept at 10 by adding a NaOH solution (0.5 M) until no NaOH consumption was observed. The oxidation reaction was stopped by the addition of approximately 100 mL of EtOH. The resulting fiber suspension was thoroughly washed with distilled water *via* vacuum filtration. Subsequently, the fiber suspension was diluted to approximately 0.2 wt% and subjected to a high-pressure water-jet system equipped with a counter-collision chamber (Star Burst, HJP-25008, Sugino Machine Co., Ltd, Namerikawa, Japan). The pulp suspension was ejected from a nozzle (with a diameter of 0.10 mm) at 245 MPa for 30 cycles, yielding a TEMPO-oxidized cellulose nanofiber dispersion. The carboxylate content of the TEMPO-oxidized cellulose nanofibers was determined through conductometric titration using an automatic titrator, as described in a previous study^47^ (AUT-701, DKK-TOA Corp., Tokyo, Japan).

### 4.3 Preparation of TEMPO-oxidized cellulose nanopapers

To prepare hexane-exchanged nanopaper, the water dispersion of the TEMPO-oxidized cellulose nanofibers (0.1 wt%, 100 mL) was vacuum-filtered (AS-01, AS ONE Corp., Osaka, Japan) through a hydrophilic polytetrafluoroethylene (PTFE) membrane filter (H020A090C, pore diameter: 0.1 μm, Advantec Toyo Roshi Kaisha, Ltd, Tokyo, Japan) for approximately 8 h. Subsequently, 100 mL of EtOH, 100 mL of EtOH, and 100 mL of hexane were poured onto a wet sheet and vacuum-filtered in that order. The resulting wet sheet was then dried under continuous vacuum filtration at room temperature for approximately 24 h, followed by peeling off from the PTFE membrane filter to obtain the hexane-exchanged nanopaper. The EtOH-exchanged nanopaper was prepared using a similar method to that of the hexane-exchanged nanopaper, except for the addition of hexane. Conventional nanopaper was prepared by drying of the water dispersion of the TEMPO-oxidized cellulose nanofibers under continuous vacuum filtration at room temperature for approximately 32 h without the addition of EtOH and hexane.

### 4.4 Evaluation of air permeability

The air permeability of the nanopapers was measured using a gas, vapor, and liquid barrier analysis system (GTR-10XF, GTR TEC Corp., Kyoto, Japan) at 23 °C, 50% RH, according to ISO 15105-2:2003. Briefly, each nanopaper was placed between two chambers. One chamber was supplied with air, whereas the other chamber was fed with a carrier helium gas at a flow rate of 40 mL min^−1^. The total pressure in both chambers was maintained at 100 kPa. In the air-supplied chamber, the air had a higher partial pressure than the other chambers, causing air to permeate the nanopaper and migrate to the other chamber fed with the carrier gas. The carrier gas conveyed the permeated air to a continuous cycle sampler and injected it into the gas chromatograph within the GTR-10XF to evaluate air permeability.

### 4.5 Evaluation of optical transparency

The total light transmittance (*T*_t_) spectra were recorded using a UV-vis spectrophotometer with an *φ*60 mm integrating sphere attachment (U-3900, Hitachi High-Tech Corp., Tokyo, Japan), while the parallel light transmittance (*T*_p_) spectra were recorded without using the integrating sphere attachment. Diffuse light transmittance (*T*_d_) was calculated using the following equation:*T*_d_(%) = *T*_t_ − *T*_p_

The haze value was calculated using the following equation:
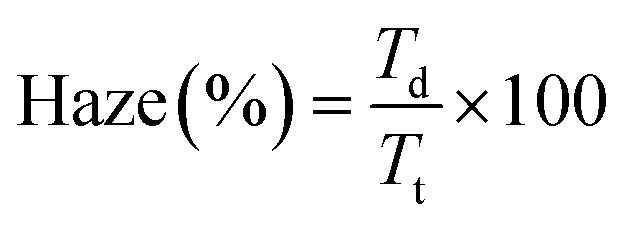


All the measurements were carried out at 23 °C and 50% RH.

### 4.6 Characterization of the porous structures

The surfaces and cross-sections of the nanopapers were observed using FE-SEM (SU-8020, Hitachi High-Tech Science Corp., Tokyo, Japan) at an accelerating voltage of 2 kV. Prior to FE-SEM observation, platinum sputter coating of the nanopapers was conducted using an E-1045 Ion Sputter (Hitachi High-Tech Science Corp., Tokyo, Japan) at a current of 20 mA for 10 s. Pore size distribution curves were obtained based on the Brunauer–Emmett–Teller and Barrett–Joyner–Halenda models using a pore size analyzer (NOVA 4200e, Quantachrome Instruments, Kanagawa, Japan). The porosity of each nanopaper was calculated using the following equation:^30^
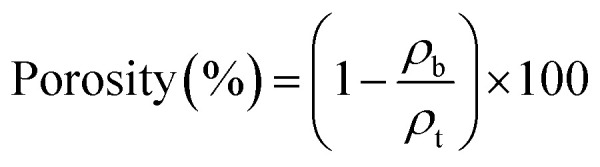
where *ρ*_b_ denotes the bulk density of the nanopapers, and *ρ*_t_ denotes the true density of the TEMPO-oxidized cellulose nanofibers with carboxylate content of 1.37 mmol g_cellulose_^−1^, which is 1.68 g m^−3^ according to a previous study.^48^

## Author contributions

Conceptualization: H. K.; methodology: Y. H. and H. K.; investigation, formal analysis, validation, and data curation: Y. H.; visualization: T. Y. and H. K.; resources: T. K., M. N., and H. K.; project administration: H. K.; funding acquisition: Y. H. and H. K.; supervision: M. N. and H. K.; writing—original draft preparation: Y. H.; writing—review and editing: T. K., M. N., and H. K.

## Conflicts of interest

The authors declare no conflicts to declare.

## Supplementary Material
